# Workshop on reconstruction schemes for magnetic resonance data: summary of findings and recommendations

**DOI:** 10.1098/rsos.160731

**Published:** 2017-02-15

**Authors:** Esin Ozturk-Isik, Ian Marshall, Patryk Filipiak, Arnold J. V. Benjamin, Valia Guerra Ones, Rafael Ortiz Ramón, Maria del C. Valdés Hernández

**Affiliations:** 1 Institute of Biomedical Engineering, Bogazici University, Istanbul, Turkey; 2 Department of Neuroimaging Sciences, Centre for Clinical Brain Sciences, University of Edinburgh, Chancellor's Building, 49 Little France Crescent, Edinburgh EH16 4SB, UK; 3 Institute of Computer Science, University of Wroclaw, Wroclaw, Poland; 4 Institute of Applied Mathematics, Delft University of Technology, The Hague, Netherlands; 5 Centre for Biomaterials and Tissue Engineering, Universitat Politècnica de València, Valencia, Spain

**Keywords:** magnetic resonance imaging, compressed sensing, super-resolution, magnetic resonance spectroscopy, image quality, image reconstruction

## Abstract

The high-fidelity reconstruction of compressed and low-resolution magnetic resonance (MR) data is essential for simultaneously improving patient care, accuracy in diagnosis and quality in clinical research. Sponsored by the Royal Society through the Newton Mobility Grant Scheme, we held a half-day workshop on reconstruction schemes for MR data on 17 August 2016 to discuss new ideas from related research fields that could be useful to overcome the shortcomings of the conventional reconstruction methods that have been evaluated to date. Participants were 21 university students, computer scientists, image analysts, engineers and physicists from institutions from six different countries. The discussion evolved around exploring new avenues to achieve high resolution, high quality and fast acquisition of MR imaging. In this article, we summarize the topics covered throughout the workshop and make recommendations for ongoing and future works.

## 1. Introduction

The era of digital revolution is driving the development of new kinds of sensing, communication and information representation systems with demands of ever-increasing fidelity and resolution. This is commonly achieved by means of compressing or reducing in some way the information acquired. The high-fidelity reconstruction of compressed and low-resolution signals has become one of the forefront areas of research nowadays on different fields, and magnetic resonance (MR) acquisition and data analysis are not exemptions. While promising results have been reported, especially in the applications of super-resolution methods [1], preliminary results on fast-acquisition (i.e. compressed sensing) techniques show that more work needs to be done prior to its application in clinics.

We held a half-day workshop on reconstruction schemes for MR data on 17 August 2016 to discuss new ideas from related research fields that could be useful to overcome the shortcomings of the conventional reconstruction methods that have been evaluated to date [2]. The discussion evolved around exploring new avenues to achieve high resolution, high quality and fast acquisition of MR imaging. The workshop was sponsored by the Royal Society through the Newton Mobility Grant Scheme. Attendees from diverse backgrounds (full list in electronic supplementary material, table S1 (online)) were from the Institute of Digital Communications, Centre for Clinical Brain Sciences, Brain Research Imaging Centre and the Compressed Sensing Group of the University of Edinburgh, the Biomedical Imaging Centre of the University of Aberdeen, the Institute of Applied Mathematics of Delft University of Technology, the Institute of Computer Science of the University of Wroclaw, the Institute of Biomedical Engineering of Bogazici University, and the Centre for Biomaterials and Tissue Engineering of Universitat Politècnica de València.

## 2. Results and discussion

**Figure 1.**
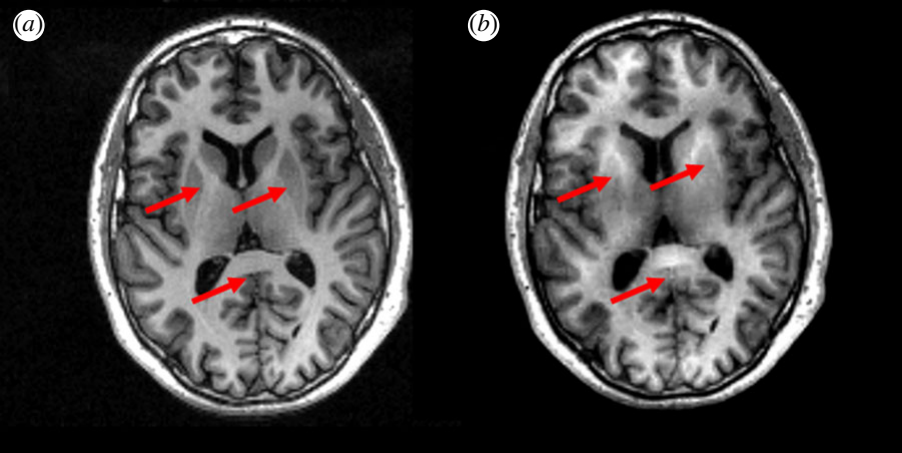
Example images from 3D inversion-recovery-prepared gradient echo scans of a healthy volunteer. Fully sampled (*a*) and four times undersampled with compressed sensing reconstruction (*b*) results are shown. Reconstruction artefacts in the undersampled scan caused apparent brightening of deep grey matter (arrows), particularly in the basal ganglia. The sampling pattern and reconstruction parameters were optimized using the mean squared error, which may not be ideal for these relatively low-contrast structures.

Compressed sensing has been a way of achieving higher resolution and/or faster MR imaging. Applications of compressed sensing to structural MR [3] and ^31^P-MR spectroscopic imaging [4] were presented and discussed. Initial evaluation on normal volunteers [3] and patients with brain tumours [5] show promising results. However, proper selection of k-space sampling pattern, validating quality of the resultant images and optimization of regularization parameters for the optimal solution of the inverse problem that would balance the fidelity to the undersampled raw data and sparsity in the transform domain have been challenging [3] (figure 1). These results were coincident with those analysed on a recent review on the use of compressed sensing in the clinical settings, which concluded that more work involving larger patient populations is needed to prove the diagnostic efficacy of compressed sensing, and that optimal imaging parameters should be determined before a wider clinical usage could be supported [2].

Recent advances in super-resolution MR may offer the possibility of improving the resolution of MR images and was mentioned as an avenue worth exploring. Efforts on novel acquisition methods for super-resolution, which have reported good results were mentioned. Ideas on post-processing existing images by means of applying super-resolution methods successfully applied to other types of images were presented and discussed.

One of these super-resolution methods, proposed by Valdés Hernández and Inamura in 2000, uses data fusion and back-propagated neural networks to enhance up to five times the resolution of satellite images [6]. Nowadays, convolutional neural networks have emerged as the optimal solution for many image analysis problems, and the idea presented by Valdés Hernández and Inamura more than 15 years ago, implemented, instead, on a convolutional neural network approach was proposed as an approach worth trying in the near future.

Other approaches to address the super-resolution issue in the context of MR imaging were presented. They are based on *sparse coding* [7] and exploit the fact that each signal *x* ∈ **R***^d^* can be represented as a linear combination *x* = *α*_1_*D*_1_ + *α*_2_*D*_2_ + ⋯ + *α_K_D_K_*, where *D* = [*D_1_ D*_2 _… *D_K_*] ∈ **R**^d^^×*K*^ is a matrix representing a so-called *dictionary,* and *α* = (*α*_1_, *α*_2_, … ,*α*_K_) ∈ **R***^K^* is a vector of real-valued coefficients, most of which are zero. In a typical scenario of the super-resolution context, the aim is to find two dictionaries *D*^h^ and *D*^l^ for the two coupled feature spaces, ***X***^h^ and **Y**^l^ (respectively), where ***X***^h^ is the space of high-resolution image patches whereas **Y**^l^ is the space of low-resolution observations of patches in ***X***^h^. It is then further assumed that the sparse representation of each *x*^h^ ∈ ***X***^h^ in terms of *D*^h^ is the same as that of its corresponding observation *y^l^* ∈ **Y**^l^ in terms of *D*^l^ [8]. Formally, the above-defined objective can be formulated as an optimization problem of the following form:

2.1 $$\underset{D^{h},D^{l},\alpha }{\min}\overset{N}{\underset{i=1}{\sum }}\left(∥x_{i}^{h}-D^{h}\alpha {∥}_{2}^{2}+∥y_{i}^{l}-D^{l}\alpha {∥}_{2}^{2}\right)+\lambda ∥\alpha {∥}_{1},$$

with ${x_{i}}^{h}\in {\text{X}}^{h}$ and ${y_{i}}^{l}\in {\text{Y}}^{l}$ being the coupled high- and low-resolution patches (respectively) for all *i* = 1, … ,*N* and a fixed *λ* > 0.

It is worth noticing that the above problem, while formulated in such a general form, is clearly not convex, hence a number of numerical approaches were proposed to handle that issue. The straight-forward technique to alternately optimize the directories *D*^h^ and *D*^l^ while assuming that the coefficients *α* are fixed and vice versa until the global optimum is eventually reached [8] was mentioned. However, a variety of contemporary methods based on computational intelligence or machine learning to speed up the optimization process [9] were also mentioned. An application of the algorithm proposed by Kato in [10] (e.g. multi-frame case), other approaches using convolutional neural networks and contemporary evolutionary algorithms were among the possible solutions presented.

Finally, an example of Graphic Unit Interface that harmonizes and combines different imaging modalities (microscopy and structural, quantitative and diffusion MRI) to explore inter-modality correspondence in regions of interest was presented [11]. Implementation of such interfaces will be useful in the present stage to help in the evaluation of the novel MR reconstruction techniques discussed. We believe that these trends in MR imaging will pick up and we will be seeing more of these studies in the near future.

As the techniques presented by the different attending sites were complementary, it was suggested that each site applies its technique to other types of data so as to allow comparability of the super-resolution and compressed-sensing methods and results between research groups. The necessity of establishing a long-term inter-site collaboration for this purpose was agreed. This will allow to identify the shortcomings of the current methodologies and set up a joint strategy for the near future.

## Research ethics

From the works presented at this workshop, only two involved the acquisition of magnetic resonance imaging from individuals. E.O.-I. obtained informed consent from the study participants and/or next-of-kin of the study participants, and approval from the Institutional Review Board for Research with Human Subjects and the Ethics Coordinating Committee (EUK) at Bogazici University, Istanbul (http://www.boun.edu.tr/en-US/Content/About_BU/Governance/Councils_Boards_and_Committees/Ethics_Committeeshttp://www.boun.edu.tr/en-US/ Content/About_BU/Governance/Councils_Boards_and_Committees/Ethics_Committees). I.M. only acquired images from one healthy volunteer, who gave written informed consent.

## Supplementary Material

Table with the full list of participants to the workshop

## Data accessibility

Primary data used on each publication referred to in this manuscript are hosted and managed locally by the respective laboratories where the work presented at this workshop was carried out and may be accessed upon request to studies' Principal Investigators at the respective institutions. All evidence discussed and presented in this manuscript has been presented/submitted previously elsewhere and can be accessed via Web of Knowledge database. The presentations/materials discussed at this workshop can be accessed at the following links: Making do with less (data): Compressed Sensing in MRI: http://hdl.handle.net/1842/18762; Optimizing calibration kernels and sampling pattern for ESPIRiT-based compressed sensing implementation in 3D MRI: http://hdl.handle.net/1842/18759; Multi-frame Super Resolution based on Sparse Coding: http://hdl.handle.net/1842/18761; Magnetic Resonance Microimaging of a Swine Infarcted Heart: Performing Cardiac Virtual Histologies: http://hdl.handle.net/1842/18763; Proactive Evolutionary Algorithms for Dynamic Optimization at the Dryad Digital Repository: http://dx.doi.org/10.5061/dryad.gg5td [12]; Considerations in applying compressed sensing to *in vivo* phosphorus MR spectroscopic imaging of human brain at 3T: http://link.springer.com/article/10.1007%2Fs11517-016-1591-9; Accelerated phosphorus magnetic resonance spectroscopic imaging using compressed sensing: http://ieeexplore.ieee.org/document/6346128/figures; Reconstruction Schemes for MR Data. Discussion Session: http://hdl.handle.net/1842/18760; Photo-gallery of the workshop: https://cil.boun.edu.tr/content/reconstruction-schemes-mr-data-workshop-university-edinburgh-england-august-17th-2016.
