# Supplementary material for: Workshop on reconstruction schemes for magnetic resonance data: summary of findings and recommendations
Source: R Soc Open Sci. 2017 Feb 15;4(2):160731. doi: 10.1098/rsos.160731 (PMC5367301; doi:10.1098/rsos.160731)
Supplement: Table with the full list of participants to the workshop [file rsos160731supp1.pdf]

Workshop on Reconstruction Schemes for MR data: Summary of Findings and Recommendations

Supporting Table S1. Full list of participants to the workshop

Name	Affiliation
Devasuda Anblagan	CCACE, University of Edinburgh, UK
Wajiha Bano	BRIC, University of Edinburgh, UK
Arnold Benjamin	BRIC, University of Edinburgh, UK
Zaid Bin Mahbub	BRIC, University of Edinburgh, UK
Martin Connell	CRIC, University of Edinburgh, UK
Michael Davies	IDCOM (Director), University of Edinburgh, UK
Patryk Filipiak	University of Wroclaw, Poland
Mohammad Golbabee	IDCOM, University of Edinburgh, UK
Victor Gonzalez-Castro	CCBS, University of Edinburgh, UK
Calum Gray	CRIC, University of Edinburgh, UK
Valia Guerra Ones	DIAM, TU Delft, The Netherlands
Jiabao He	Aberdeen Biomedical Imaging Centre, UK
Christina Lemke	CMVM, University of Edinburgh, UK
Ross Lennen	CCBS, University of Edinburgh, UK
Ian Marshall	CCBS, University of Edinburgh, UK
Rafael Ortiz Ramón	Universitat Politècnica de València, Spain
Esin Ozturk Isik	Bogazici University, Turkey
Enrico Pellegrini	CCBS, University of Edinburgh, UK
Michael Thrippleton	BRIC, University of Edinburgh, UK
Maria del C. Valdés Hernández	CCBS, University of Edinburgh, UK
Mehrdad Yaghoobi	IDCOM, University of Edinburgh, UK

Note: BRIC: Brain Research Imaging Centre, CRIC: Clinical Research Imaging Centre, CCBS: Centre for Clinical Brain Sciences, IDCOM: Institute for Digital Communications, CCACE: Centre for Cognitive Ageing and Cognitive Epidemiology, CMVM: College of Medicine and Veterinary Medicine
